# Oviductal Transcriptome Is Modified after Insemination during Spontaneous Ovulation in the Sow

**DOI:** 10.1371/journal.pone.0130128

**Published:** 2015-06-22

**Authors:** Rebeca López-Úbeda, Francisco A. García-Vázquez, Raquel Romar, Joaquín Gadea, Marta Muñoz, Ronald H. F. Hunter, Pilar Coy

**Affiliations:** 1 Department of Physiology, Veterinary Faculty, University of Murcia, Murcia, Spain; 2 International Excellence Campus for Higher Education and Research (Campus Mare Nostrum), Murcia, Spain; 3 IMIB-Arrixaca (Institute for Biomedical Research of Murcia), Murcia, Spain; 4 Centro de Biotecnología Animal—SERIDA, Deva, Gijón, Asturias, Spain; 5 Sidney Sussex College, Cambridge, England; Clermont-Ferrand Univ., FRANCE

## Abstract

Gene Expression Microarray technology was used to compare oviduct transcriptome between inseminated and non-inseminated pigs during spontaneous oestrus. We used an *in vivo* model approaching the study from a physiological point of view in which no hormonal treatment (animals were in natural oestrus) and no artificial sperm selection (selection was performed within the female genital) were imposed. It is therefore emphasised that no surgical introduction of spermatozoa and no insemination at a site other than the physiological one were used. This approach revealed 17 genes that were two-fold or more up-regulated in oviducts exposed to spermatozoa and/or developing embryos and 9 genes that were two-fold or more down-regulated. Functional analysis of the genes revealed that the top canonical pathways affected by insemination were related to the inflammatory response and immune system (Network 1) to molecular transport, protein trafficking and developmental disorder (Network 2) and to cell-to-cell signalling and interaction (Network 3). Some of the genes in network 1 had been previously detected in the oviduct of human and animals, where they were over-expressed in the presence of spermatozoa or pre-implantation embryos (*C3*, *IGHG1*, *ITIH4*, *TNF* and *SERPINE1*) whereas others were not previously reported (*SAA2*, *ALOX12*, *CD1D* and *SPP1*). Genes in Network 2 included *RAB1B* and *TOR3A*, the latter being described for the first time in the oviduct and clearly expressed in the epithelial cells of the mucosa layer. Network 3 integrated the genes with the highest down-regulation level (*CYP51*, *PTH1R* and *TMOD3*). Data in the present study indicate a change in gene expression during gamete encounter at the site of fertilization after a natural sperm selection within the female genital tract. These changes would indicate a modification of the environment preparing the oviduct for a successful fertilization and for an adequate embryo early development.

## Introduction

The oviductal transcriptome has attracted special interest in recent years because subtle changes in gene expression around the time of fertilization or shortly thereafter could be related to infertility, abnormal development or even health problems in adulthood [1]. The development of analytical tools such as microarrays has enabled to generate a complete database for transcript changes in the oviduct at fertilization time [2]. To date, various studies have analysed changes in specific oviductal transcripts under different experimental conditions [3–8]. By contrast, a body of work in different species has focused on the endometrial response to the presence of young embryos [9–14].

The pig is a species of great commercial interest in which the development *in vitro* of assisted reproductive technologies is held back by its susceptibility to extensive polyspermy upon *in vitro* fertilization compared with other species [15,16]. This differs dramatically from *in vivo* fertilization which is highly efficient due to the effectiveness of a series of natural barriers to polyspermy [15,17]. For this vital reason amongst others, increased knowledge of molecular pathways in the oviduct contributing to successful fertilization could lead to a significant advance in developments for the commercial exchange of porcine *in vitro* produced embryos or for biomedical purposes (e.g. transgenesis, cloning or xenotransplantation).

Previous studies have described the proteomic changes in the pig oviduct mediated by the presence of gametes in genital tracts collected at slaughterhouses [18] or in animals undergoing surgical intervention [8,19], but there is still a lack of information concerning the complete transcriptomic profile of this organ in fertile sows in conditions close to physiological. The hormonal induction of ovulation, used in some experimental designs, alters the physiological pathways leading to gamete encounter and a number of immature oocytes can be found in the oviductal ampulla after such treatments [20–22]. Direct insemination of spermatozoa into the pig oviduct produces polyspermy [23,24] and surgical interventions can induce inflammatory responses, thus altering the transcriptome of a specific tissue [25], leading to erroneous or contradictory outcomes when experimental designs involving some of these processes are used.

Although specific targeted genes have been analysed by real time quantitative polymerase chain reaction (RT-qPCR) [8], and microarray technology has been used recently in one experiment involving surgical insemination of sex-sorted spermatozoa directly into the oviduct [19], no data are available concerning the effect of gametes or zygotes during the very early stages of fertilization on the porcine oviductal transcriptome in conditions resembling physiological situation. The question therefore arose as to whether the meeting of male and female gametes in the oviduct could influence the transcriptome.

The objective of this study was to investigate differences in oviductal transcriptome between inseminated and non-inseminated pigs during spontaneous oestrus in a specific area of the oviduct (ampullary-isthmic junction). We decided to analyse this specific part of the oviduct where spermatozoa released from sperm reservoir arrive close to the time of ovulation because it is where fertilization and zygote formation occurs [26].

We used an *in vivo* model approaching the study from a physiological point of view in which no hormonal treatment (animals were in natural oestrus) and no artificial sperm selection (selection was performed within the female genital tract after cervical sperm deposition) were imposed. It is therefore emphasised that no surgical introduction of spermatozoa and no insemination at a site other than the physiological one were used. The principal tool used to achieve our objective was the Porcine Gene Expression Microarray (ID 026440, Agilent Technologies, Madrid, Spain).

## Materials and Methods

### Animals

This study was carried out in strict accordance with the recommendations in the Guiding Principles for the Care and Use of Animals (DHEW Publication, NIH, 80–23). The protocol was approved by the Ethical Committee for Experimentation with Animals of the University of Murcia, Spain (Project Number: 11996/PI/09). Surgery was performed under analgesic and anaesthetic protocols [27], and all efforts were made to minimize suffering.

Ten cyclic commercial hybrid sows Landrace x Large White were kept under standard conditions of housing and feeding. All were in lactation at the same time and no hormonal induction or surgery (before obtaining the samples) was performed in the animals of this study.

### Oestrous detection, artificial insemination and tissue collection

**Fig 1:**
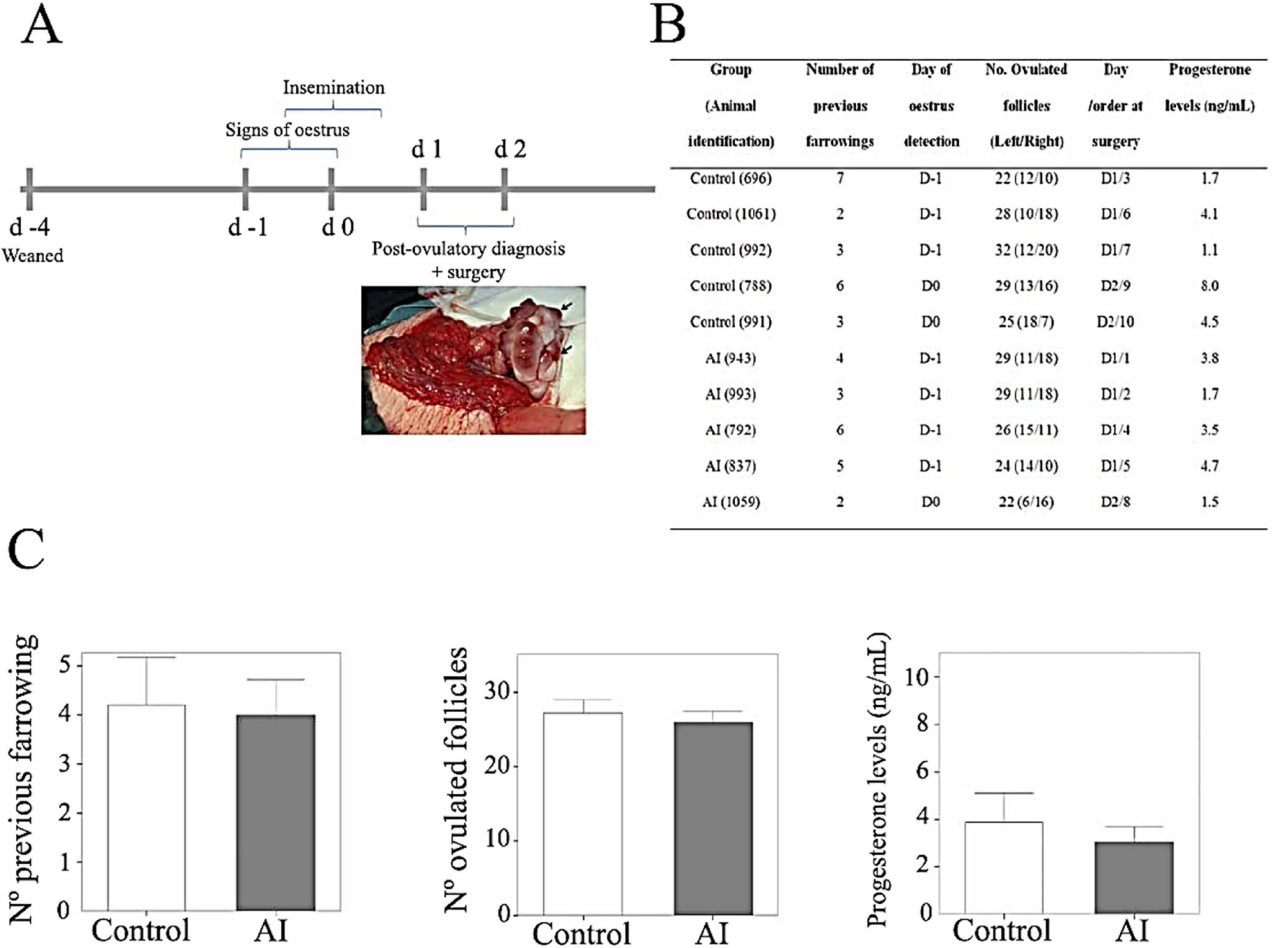
(A) Representative diagram of the experimental protocol for the recovery of the oviductal tissue samples and image of the left ovary of sow number 5, showing the recently ovulated follicles as corpora haemorrhagica (arrows). **(B)** Animal dataset. **(C)** Comparative histograms of physiological data for inseminated and non-inseminated animals.

The animals were weaned on day -4 (Fig 1A). Five animals were artificially inseminated (AI) ≈10 hours after showing signs of standing oestrus (day -1 or 0; Fig 1A), whereas the remaining 5 were not inseminated (Control). Previous ultrasonographic scanning was performed to ensure the presence of growing follicles (8–12 mm diameter) in both ovaries. A diagram of the experimental design is shown in Fig 1A and animal data sheet is shown in Fig 1B.

Inseminations were performed with seminal doses from mature hybrid boars of proven fertility. Fresh semen diluted in 100 ml of Beltsville Thawing solution (BTS, [28]) at a concentration of 3 x 10^7^ spermatozoa/ml was used.

Animals were diagnosed as post-ovulatory when no large follicles were observed by ultrasonography (on day 1 or 2), ensuring that ovulation had occurred before removing the oviducts. Then, animals were subjected to sedation and anaesthesia in order to perform a mid-ventral incision and expose the genital tract. The ovaries were photographed, the number of recently-ovulated follicles was recorded (Fig 1B and 1C), and the oviducts were removed no more than 20 minutes after sedation. No differences between inseminated and control groups were found for the number of previous farrowings, the number of ovulated follicles nor for progesterone concentrations (Fig 1C; p> 0.05). These data are similar to those detected in previous studies by other authors [29,30].

Complete oviductal tissue samples from the ampullary-isthmic junction (sample size >1cm including the end of the ampulla) were taken and immediately frozen in liquid nitrogen for mRNA extraction, cDNA reverse transcription and further study by microarray. Oviductal flushing was not performed to avoid transcriptome alteration. Tissue samples were fixed immediately after collection in Bouin’s solution for the immunohistochemical assay. Blood samples were also taken in order to determine the systemic oestradiol and progesterone concentrations for each animal (Fig 1B), using a chemoluminescence immunoassay of microparticles (ARCHITECT, Abbot, IL).

### RNA isolation

Total RNA was extracted using the ‘TRIzol method’ according to the protocol recommended by the manufacturer (Life Technologies Inc., Gaithersburg, MD). The quantity and quality of the RNA samples were analysed with ARN 6.000 NanoLabChip kit and the Agilent 2100 Bionalyzer (Agilent Technologies, Santa Clara, CA) respectively. Values of RNA integrity number (RIN) in analysed samples ranged from 6.5 and 8.5, and only those samples with RIN > 7.0 were used for microarray analysis.

### Microarray analysis

Equal amounts of total RNA (300 ng) from samples of the oviducts of each animal were used. All the samples were hybridized in the Porcine Gene Expression Microarray (ID 026440 Agilent Technologies, Madrid, Spain) which encompassed 43,803 probes. Protocols for sample preparation and hybridization of the oviductal samples were adaptations of those in the Agilent Technical Manual. In brief, first strand cDNA was transcribed from 300 ng of total RNA using T7-Oligo (dT) Promoter Primer. Samples were transcribed *in vitro* and Cy-3-labelled using a Quick-AMP labelling kit (Agilent Technologies, Madrid, Spain). The synthesis yielded 10–15 μg of cRNA. Following a further clean-up round (Qiagen), the cRNA was fragmented into pieces ranging from 35 to 200 bases in size, and confirmed using Agilent 2100 Bioanalyzer technology. Fragmented cRNA samples (1.65 μg) were hybridized onto chips by incubating for 17 h at 65.8°C with constant rotation, followed by a two-step microarray wash of 1 min in two buffers (Agilent Technologies, Madrid, Spain). Hybridized microarrays were scanned in a GenePix 4100A scanner (Molecular Devices, Sunnyvale, CA).

The Gene Pix Pro 6.0. software was used for array image analysis and for the calculation of spot intensity measurements, which were considered to be raw data. Statistical analysis was performed using BioConductor for R with the packages Linear Models for Microarray data (LIMMA), Marray, pcaMethods, EMA and RankProd. The data were analysed by subtracting the background using the “Normexp” method, from the LIMMA package, with an offset of 10. Finally they were normalized by the “quantile” method form the same LIMMA package. Cut-off values were set to a two-fold difference in expression values and a false discovery rate of 1%. Identified probe sets were compared with published sequence databases using the basic local alignment search tool at the National Centre for Biotechnology Information (http://www.ncbi.nlm.nih.gov/blast/blast.cgi). If a gene was identified in the results by more than one probe set, the mean fold change was calculated.

All results from microarray analysis were submitted to Gene Expression Omnibus database with the accession number GSE68148.

### Ingenuity pathway analysis

To integrate our results into a more general model, differentially expressed transcripts classified according to Gene Ontology (GO) and functional associations were investigated using the Ingenuity Pathway Analysis software (IPA, Ingenuity Systems, Redwood City, CA). As indicated, only genes with a fold change >2 (at least in one of the probe sets where the gene was identified) were included. Moreover, Osteopontin (*SPP1*) with a fold change of 1.47 was also included in the Ingenuity analysis because previous studies had shown its specific role in porcine fertilization [31].

### Microarray validation by Real-time RT-PCR

The relative expression levels of eight selected genes in the total RNA from the oviduct were determined by real-time RT-PCR using specific primers for each gene (Table 1): torsin 3A (*TOR3A*), Ras-related protein Rab-1B (*RAB1B*), arachidonate 12-lipoxygenase (*ALOX12*), glutathione S-transferase alpha 1 (*GSTA1*), complement component 3 (*C3*), inter-alpha-trypsin inhibitor heavy chain family, member 4 (*ITIH4*), Osteopontin (*SPP1*) and tropomodulin 3 (*TMOD3*). *β-actine* and *GADPH* genes were used as control for the data normalization. The qPCR experiment was performed as follows. First, the retrotranscription reaction was made using MultiScribe Reverse Transcriptase (Life Technologies, Inc.) and 1 μg of complete RNA. After this, the qPCR was completed in a StepOne Thermal Cycler (Applied Biosystems, Foster City, CA) using the 5x HOT FIREPolEvaGreenqPCR Mix Plus (Solis BioDyne, Tartu, Estonia), with SYBR Green and normalizing with ROX. The Cts values were obtained from the OneStep Software. The genomic DNA contamination was abolished using a DNAse treatment and oligonucleotides designed with their sequence interrupted by intronic regions. The Cts values were analysed by statistical software, comparing the average between inseminated and non-inseminated samples, and obtaining the fold change values. The p-value was calculated between both groups and in all cases the significance was statistically relevant (p-value < 0.05).

**Table 1:** Primer sequences used to amplify specific fragments of porcine transcripts.

Gene symbol	Gene Bank accession	PCR-product (bp)	Melting point (°C)	Forward Primer sequence	Reverse Primer sequence
*TOR3A*	AK395388	110	83.7	CAT CTT TCT TTT TCT CAG TAA CCT T	GGC TCC AAA TTT TCC ATC CT
*RABB1*	BX916033	68	82.2	TGGCATCATCGTGGTGTATGA	CCTGCAGCCACTGCTTCA
*ALOX12*	NM_213931	97	84.7	CAGGCTTGGTGTCGAGAGTT	AGTGGCAGAGCTGTTCCTTG
*GSTA1*	NM_214389	79	80.0	GATTAAATAAAGCCGGCTGAGTGT	GGTCAAGCAGTTGATTGATAGCA
*C3*	NM_214009	85	83.3	CGCCACCAACAGACTCTAACG	CCTGGAGGCCAGTCTTCAAG
*ITIH4*	NM_001001537	112	83.5	GAGTGGTCAACAAGGGCAGT	CCTGGGTAGGTCACACCATC
*SPP1*	NM_214023	78	82.9	GGACACGGACTCCGAGGAA	TCGGATTCATCGGAGTGATG
*TMOD3*	NM_214294	70	78.8	CTCAACAGGGACCTCGAACC	CGCCTCTTGCGAACTAAGTC

### Immunohistochemistry

Torsin family 3, member A (TOR3A) and GSTA1 protein expression were analysed immunohistochemically in the oviduct. The oviductal tissue samples were fixed immediately after collection in Bouin’s solution for 24 h and washed in 75% v/v methanol for 48 h. Samples were then dehydrated, embedded in a paraffin block, sectioned transversely to a thickness of 10 μm and mounted on poly-L-lysine coated slides. Tissues sections were dewaxed in xylene three times and rehydrated in a series of solutions of graded ethanol concentrations (100%, 95% and 70%) followed by distilled water. Antigen retrieval was performed by boiling the sections in citrate buffer (pH 6) for 30 minutes. To block endogenous peroxidase activity, the sections were incubated for 20 min in 80% methanol solution containing 2% hydrogen peroxide. After washing with TBS-Tween, sections were then incubated for 90 min in 6% normal goat serum (TBS-Tween) at room temperature to saturate any sites for nonspecific binding of proteins. Sections were incubated with anti-TOR3A (PAG629Hu01, Uscn Life Sciences, Wuhan, China) or anti-GSTA1 (PAA609Hu01, Uscn Life Sciences, China) primary antibodies (1:100, in TBS-Tween) overnight at 4°C. TOR3A and GSTA1 immunolocalisation was established with the VECTASTAIN ABC kit (following the manufacturer’s instructions; Vector Laboratories, Burlingame, CA). Finally, the sections were visualized using 3,3-diaminobenzidine hydrochloride (DAB Kit, AbcamPlc, Cambridge, UK), counterstained with haematoxylin, dehydrated (70%, 95% and 100% ethanol), cleared through xylene twice for 5 min and mounted (Eukitt, Sigma, Madrid, Spain). Negative controls were performed following the same procedure without primary antibodies. Immunostained sections were examined using a Nikon Eclipse bright-field microscopy and captured by NIS-Elements Br Microscope Imaging Software 3.2 (Nikon, Tokyo, Japan).

Immunohistochemical quantification was performed with Leica QWin Pro program (S1 Fig). Each pixel from digital image consisted of a number between 0 (white) and 255 (black) representing the intensity of transmitted light or grey level at a point. Grey level was related with protein content (darkest corresponded to highest protein content).

### Oviductal fluid collection

Oviducts from animals (inseminated 24h before or non-inseminated) at the early luteal phase were obtained. The fluid was collected by mechanical aspiration using an automatic pipette [32]. A mean volume of 40 μl per oviduct was collected. The oviductal fluid was then centrifuged at 7,000 g for 10 min at 4°C in order to remove cellular debris. Supernatant was kept at -80°C until use. The oviductal fluid of a total of 6 animals per group (inseminated and non-inseminated) was collected and pooled in to two batches of 3 animals each.

### SDS-PAGE and western blotting

Protein concentrations of the samples were measured by the Bradford protein assay. Samples were re-suspended in Laemmli buffer, and boiled for 3 min. After centrifugation, 1 μl of β-mercaptoethanol was added to the samples, which were boiled again for 3 min. Finally, the samples were centrifuged (14,000 *g*, 5 min, 4°C). Eighteen μg of total protein were loaded per line and subjected to SDS-PAGE and electro-transferred to PVDF membranes. The membranes were blocked with 5% BSA (A-9647, Sigma-Aldrich, Madrid, Spain) in TBS containing 0.1% Tween 20 (T-TBS). The primary antibody (anti-TOR3A, PAG629Hu01, Uscn Life Sciences, Wuhan, China) was diluted 1:400 in T-TBS + 5%-BSA. TOR3A protein was detected by secondary antibody (Rabbit anti-goat-IgG-HRP, AP106P, Millipore) diluted 1:10,000 in T-TBS + 5%-BSA and developed using an enhanced chemiluminescence detection kit (ECLplus, Amersham, GE Healthcare) according to the manufacturer’s instructions.

## Results and Discussion

The oviduct plays an important role during the reproductive process. In many mammals but even more so in pigs, it provides of a series of natural barriers against polyspermy [16,17]. Fertilization is a highly synchronized process between male and female gametes, which takes place in a very specific area of the oviduct. In pigs, it occurs in the ampullary-isthmic junction [26]. Understanding the mechanisms that act during this time would be of great importance to advance assisted reproduction techniques. In this study, we offer a new physiological perspective and provide new insights in to local changes in gene expression that take place in the porcine oviduct around the time of fertilization (sperm arrival, sperm-oocyte interaction and zygote formation).

**Fig 2:**
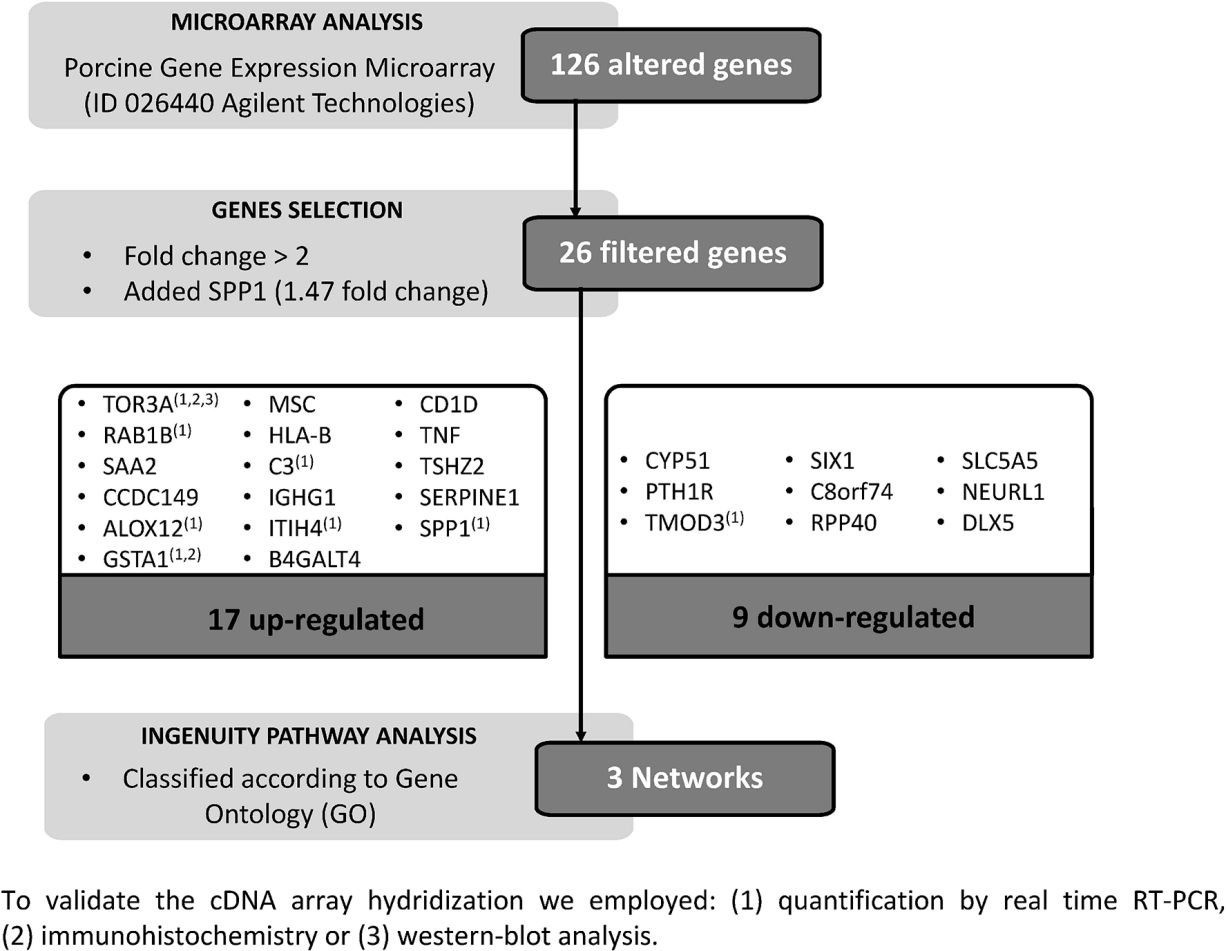
Scheme of the protocol followed to process and analysed the data obtained from array hybridization.

**Fig 3:**
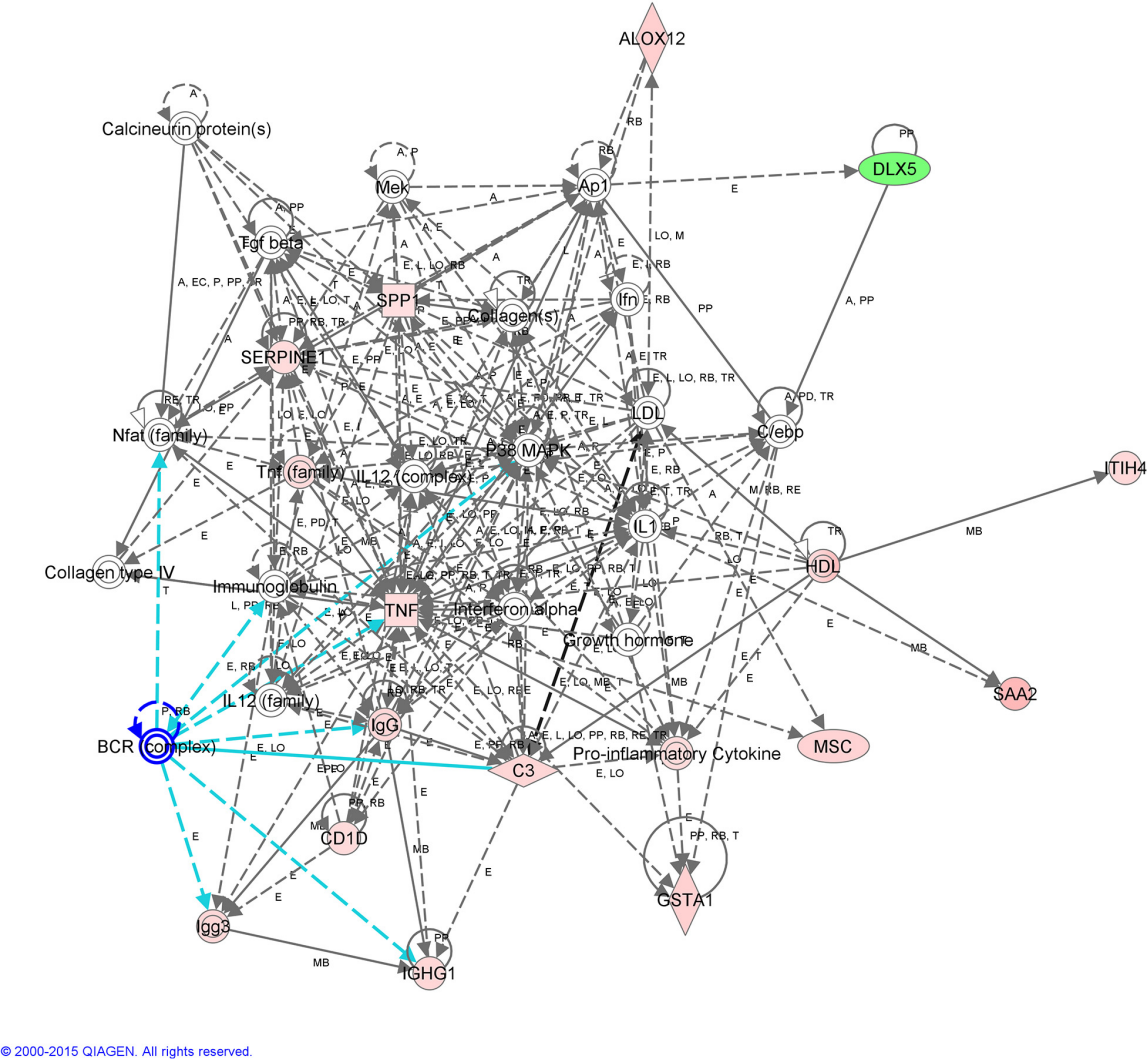
Interactome of functional associations among genes included in Network 1 by Ingenuity Pathway Analysis.

**Fig 4:**
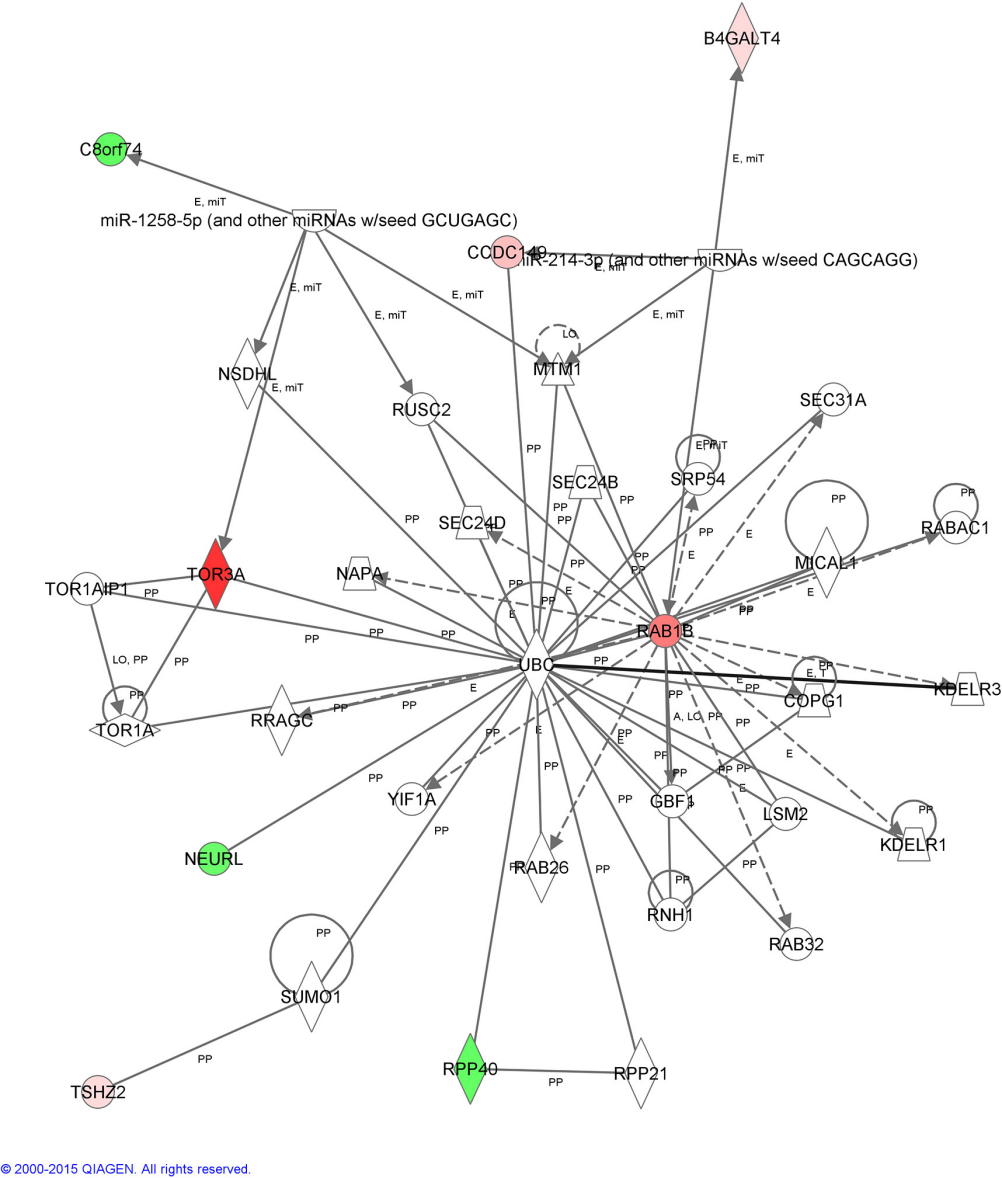
Interactome of functional associations among genes included in Network 2 by Ingenuity Pathway Analysis.

**Fig 5:**
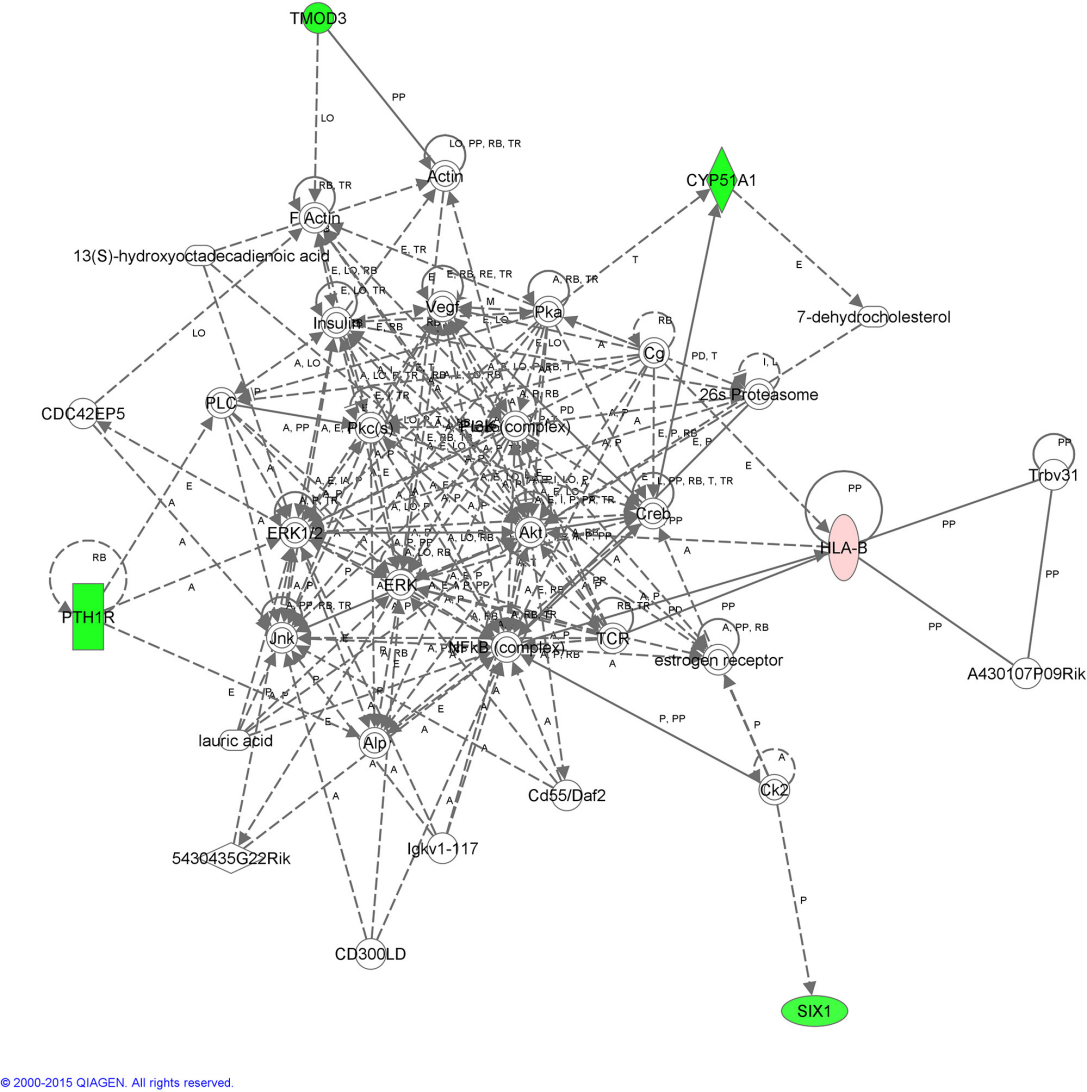
Interactome of functional associations among genes included in Network 3 by Ingenuity Pathway Analysis.

When microarray analysis was performed (Fig 2), the data analysis revealed a total of 126 genes differentially expressed (0.28% of the 43803 transcripts analysed), between the inseminated and non-inseminated sows. Setting the fold change to 2, 26 identified differentially expressed genes (DEGs) were selected. Only one gene (SPP1) with a fold change lower than 2 (1.47), was included in our analysis given its importance in reproduction (Fig 2). To integrate our microarray results into a more general model, these genes, 17 up-regulated (65.4%) (Table 2) and 9 down-regulated (34.6%) (Table 3), were classified according to Gene Ontology (GO) and their functional associations were further investigated using the Ingenuity Pathway Analysis (IPA). From this analysis, 3 networks (Figs 3–5) with a score of 27, 18 and 10 respectively were obtained.

**Table 2:** Genes up-regulated in oviduct in artificially inseminated sows.

Gene Bank accession	Gene Symbol	Entrez Gene Name	Fold Change	IPA network
AK395388	TOR3A	torsin family 3, member A	11.16	2
BX916033	*RAB1B*	RAB1B, member RAS oncogene family	7.18	2
NM_001044552	*SAA2*	serum amyloid A2	3.82	1
LOC100621948	*CCDC149*	coiled-coil domain containing 149	3.48	2
NM_213931	*ALOX12*	arachidonate 12-lipoxygenase	2.58	1
NM_214389	*GSTA1*	glutathione S-transferase alpha 1	2.51	1
AK350490	*MSC*	musculin	2.41	1
NM_001097427	*SLA-3*	MHC class I antigen 3	2.33	3
NM_214009	*C3*	complement component 3	2.20	1
LOC396781	*IGHG1*	IgG gamma 1 heavy chain	2.18	1
NM_001001537	*ITIH4*	inter-alpha-trypsin inhibitor heavy chain family, member 4	2.07	1
AY253339	*B4GALT4*	UDP-Gal:betaGlcNAc beta 1,4- galactosyltransferase, polypeptide 4	2.00	2
NM_001102680	*CD1D*	CD1d molecule	1.94	1
NM_214022	*TNF*	tumor necrosis factor	1.94	1
TC534257 (TIGRID)	*TSHZ2*	Teashirt Zinc Finger Homeobox	1.94	2
NM_213910	*SERPINE1*	serpin peptidase inhibitor, clade E (nexin, plasminogen activator inhibitor type 1), member 1	1.92	1
NM_214023	*SPP1*	Osteopontin	1.47	1

**Table 3:** Genes down-regulated in oviduct in artificially inseminated sows.

Gene Bank accession	Symbol	Entrez Gene Name	Fold Change	IPA network
NM_214432	*CYP51*	cytochrome P450, family 51, subfamily A, polypeptide 1	-3.16	3
NM_214382	*PTH1R*	parathyroid hormone 1 receptor	-3.13	3
NM_214294	*TMOD3*	tropomodulin 3 (ubiquitous)	-3.05	3
NM_001199718	*SIX1*	SIX homeobox 1	-2,66	3
XM_001924317	*C8orf74*	Chromosome 8 open Reading frame 74	-2.22	2
AK233279	*RPP40*	ribonuclease P/MRP 40kDa subunit	-2.18	2
NM_214410	*SLC5A5*	solute carrier family 5	-2.16	-
XM_001926877	*NEURL1*	neuralized homolog (Drosophila)	-2.11	2
NM_001159660	*DLX5*	distal-less homeobox 5	-1.91	1

**Fig 6:**
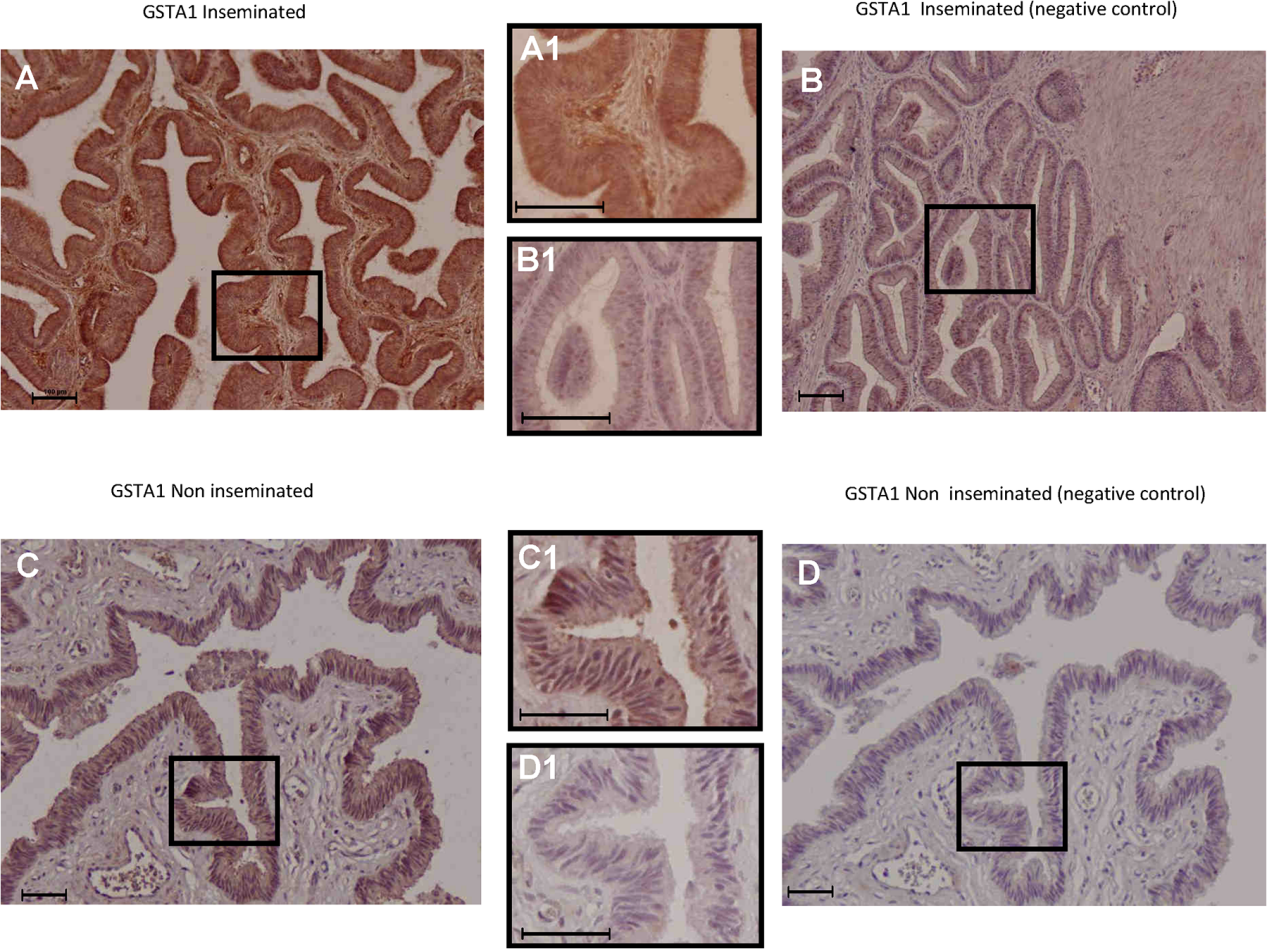
Immunohistochemical localization of GSTA1 in the porcine oviduct. GSTA1 protein expression extended from the epithelial cells to deeper layers of the oviductal wall in the inseminated sows (Fig 6A, magnified in 6A1) compared with the non-inseminated animals, where labelling was mainly observed at the epithelial level (Fig 6C, magnified in 6C1). The corresponding negative controls are shown in Fig 6B, magnified in 6B1 for the inseminated animals and in Fig 6D, magnified in 6D1, for the non-inseminated sow. In all figures, scale bars correspond to 100 μm.

**Fig 7:**
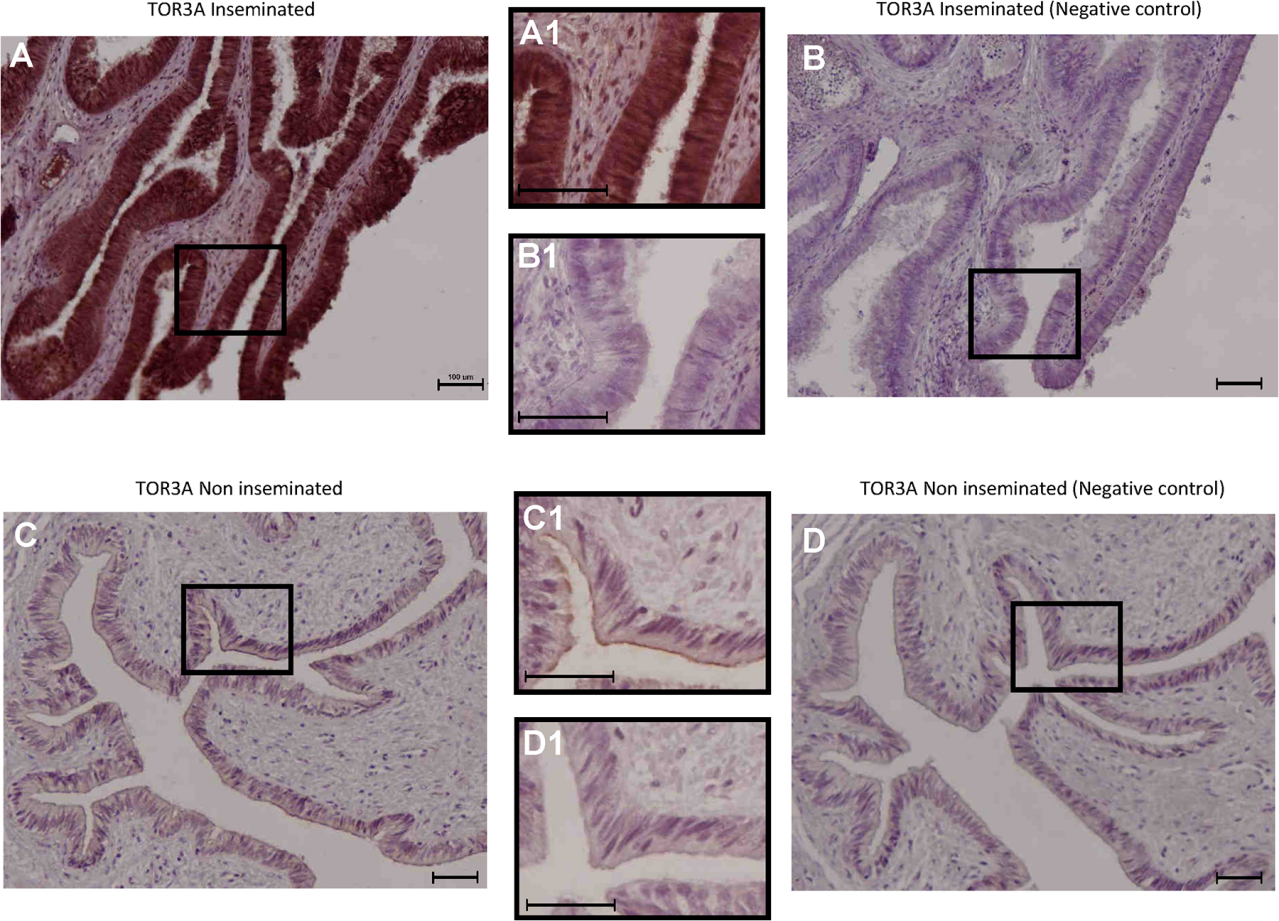
Immunohistochemical localization of TOR3A in the porcine oviduct. Strong immunostaining for TOR3A protein was only observed in the oviductal wall of the inseminated sows (Fig 7A, magnified in 7A1). The labelling was absent in non-inseminated animals (Fig 7C magnified in 7C1) as well as in the corresponding negative controls for the inseminated (Fig 7B, magnified in 7B1) and non-inseminated sows (Fig 7D, magnified in 7D1). In all figures, scale bars correspond to 100 μm.

**Fig 8:**
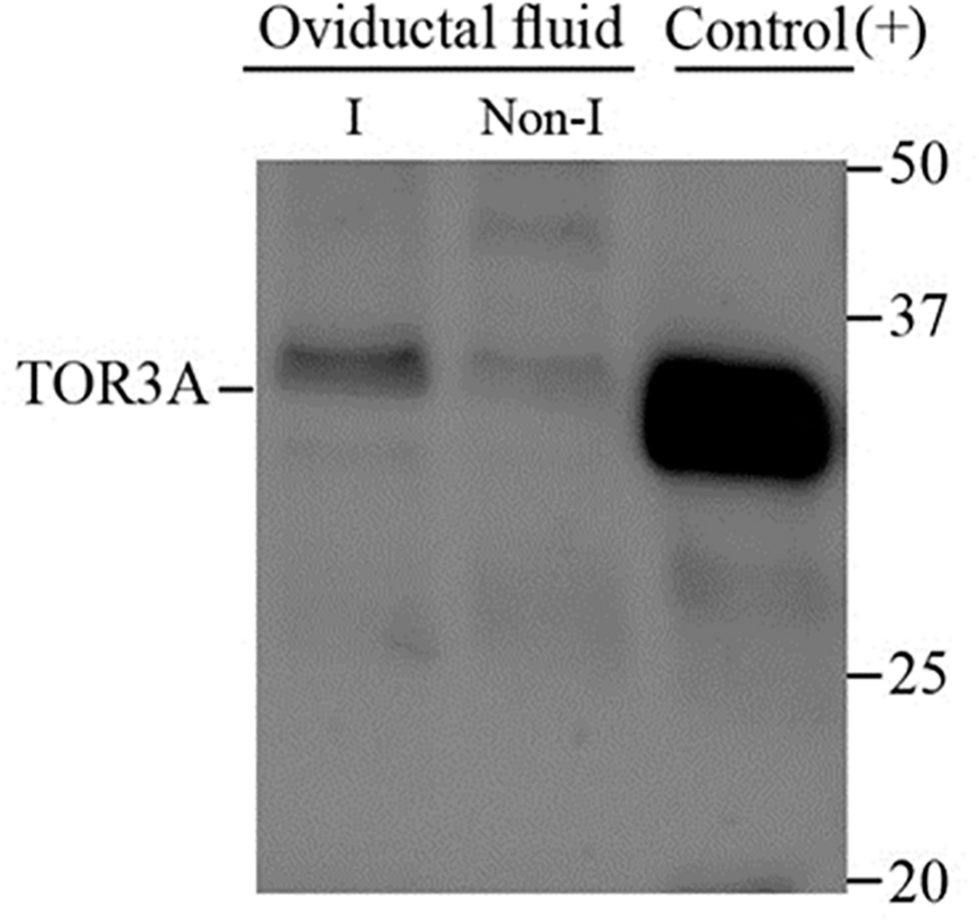
Western-blotting detection of TOR3A in porcine oviductal fluid. Expression of the TOR3A increases in the oviductal fluid of the inseminated sows (I) (Lane 1) compared to non-inseminated animals (Non-I) (Lane 2). Protein TOR3A (RPG629Hu01, Uscn Life Sciences, Wuhan, China) was used as a positive control (Lane 3). Molecular weight is expressed in kDa.

Significant gene expression differences derived from microarray analysis were validated by real-time RT-PCR for 8 genes (*TOR3A*, *RAB1B*, *ALOX12*, *GSTA1*, *C3*, *ITIH4*, *SPP1* and *TMOD3*; Table 4, p<0.05). The analysis of the same RNA samples as used for array hybridization allowed a direct comparison of both methods. We further confirmed GSTA1 and TOR3A protein expression in the oviduct by immunohistochemistry (Figs 6 and 7) and the detection of TOR3A by Western blotting into the oviductal fluid (Fig 8).

**Table 4:** RT-PCR confirmation of microarray.

	MICROARRAY		qPCR GAPDH		qPCR B-ACTINE	
Genes	Mean fold changes	P value	Mean fold changes	P value	Mean fold changes	P value
*TOR3A*	11.16	0.0001	1.33	0.031	1.47	0.038
*RABB1*	7.18	0.0001	1.38	0.029	1.34	0.056
*ALOX12*	2.58	0.0486	3.38	0.030	3.11	0.050
*GSTA1*	2.51	0.0038	1.76	0.020	1.68	0.031
*C3*	2.20	0.0188	3.89	0.001	3.79	0.001
*ITIH4*	2.07	0.0297	3.46	0.005	3.36	0.001
*SPP1*	1.47	0.0444	3.68	0.010	3.83	0.019
*TMOD3*	-3.05	0.0001	-5.06	0.008	-5.53	0.010

It is known that the transcriptome of the oviduct is affected by the presence of oocytes [6], spermatozoa [19,33], oestrous cycle stage [7,34] and surgical interventions [25]. Of special significance in our study is that the experimental design would not have acted to compromise the response observed in the oviduct tissue. Furthermore, only animals with ovulations on both ovaries (Fig 1B) were used, to ensure the presence of oocytes in both oviducts, so that the response in this study was due to the presence of both gametes at the same time in the oviduct (different to that found with gametes separately) and during their interaction to form the zygote.

Another important point to consider in our study is the sperm presence in the sampling area (ampullary-isthmic junction). In previous studies, oviducts or epithelial cells were exposed to far more than the physiological number of spermatozoa at the site of fertilization after natural mating [8,19]. Also, direct insemination of spermatozoa into the pig oviduct produces polyspermy [23,24]. The experimental design in this study attempted to simulate physiological conditions, where most spermatozoa (>70%) are eliminated in the uterine lumen [35], either by reflux (25–45%) [36] or by the action of polymorphonuclear leucocytes [37,38] during the passage along the reproductive tract, and only a small proportion of spermatozoa reach the oviduct [39,40]. In the present study, the spermatozoa were selected within the female genital tract during their ascent to the site of fertilization. Moreover, the relatively small number of highly selected spermatozoa which arrive at the sperm reservoir in the oviductal isthmus may diminish immune responses that a large amount of sperm can produce [36], thus reducing the number of activated genes related to inflammatory and immune responses in this anatomical region found in the present study.

In our study the presence of zygotes (together with sperm arrival and sperm-oocyte interaction) could be influencing the gene regulation in the ampullar-isthmic section of the oviduct. Little is known about early embryo stage and maternal communication. In a previous report [14] it was demonstrated the down-regulation of immune response genes by the presence of embryos in different stages (from 2-cell to blastocyst stage) in the oviduct and in the uterine horn, suggesting a modulation of the uterine milieu to further develop the embryos properly. Whereas the release of inflammatory cytokines by the recently formed zygote was demonstrated some years ago [41], the relationship between inflammation and the arrest or progress of the zygote and young embryos through the oviduct is worth consideration.

The seminal plasma contains several proteins and agents which influences the sperm survival and modulation. Moreover it is known that seminal plasma influences the endometrium activating changes in gene expression [42] and induces oviductal synthesis of growth factors and cytokines which support further embryo development [43]. In the present paper the insemination was performed using the ejaculated from fertile boars. However, the ejaculated, as mentioned in the material and methods section, was diluted in BTS previous to insemination (as commonly happens in commercial farms), so the level of seminal plasma in the insemination dose is very low and in consequence the impact of this fluid in the oviduct may be very limited.

Limiting our study to this area may have contributed to the lower number of genes identified compared with previous studies in which the authors analysed the entire oviduct [8,19]. There are changes in the proportions of secretory and ciliated cells between the different parts of the oviduct [44], so differences at the level of gene expression would be anticipated.

The IPA software clustered 12 of the DEGs identified in Network 1 (Fig 3). The top canonical pathway affected by AI is related to the inflammatory response and immune system. Some of these genes have been previously detected in the oviduct of human and animals (pigs and mice) where they were over-expressed in the presence of spermatozoa or pre-implantation embryos (*C3*, *IGHG1*, *ITIH4* [18], *TNF* [14] and *SERPINE1*[45]) whereas others were not previously reported (*SAA2*, *ALOX12*, *CD1D* and *SPP1*). *GSTA1* gene was also included in Network 1 (Fig 3); GSTA1 is an enzyme with glutathione peroxidase activity. Spermatozoa are highly sensitive to oxidative stress and were the first cell type in which ROS production was biochemically confirmed [46]. By up-regulating the expression of *GSTA1*, the response of oviductal tissues could keep the redox events in the surrounding environment within physiological ranges and protect spermatozoa and/or embryos from ROS-induced damage [47,48]. Immunohistochemical data confirmed that GSTA1 protein was expressed in the oviduct epithelial cells of inseminated and non-inseminated sows. The immunoreactivity detected above was stronger in the inseminated sows compared to the non-inseminated sows. In addition, in the inseminated sows (Fig 6), a moderate degree of reactivity was detected in the connective tissue of the lamina propria of the mucosa folds (Fig 6A1). However, no positive staining was observed in this histological structure in the non-inseminated sows (Fig 6C1).

The top canonical pathway associated with the DEGs clustered in Network 2 (Fig 4), are related to molecular transport, protein trafficking and developmental disorder. *TOR3A* and *RAB1B*, the two genes with the highest positive fold change of this study were found in this network. *RAB1B* is required for clearance of ubiquitinated protein aggregates [49], and the boar sperm acrosome shows a high level of de-ubiquitinating activity (ubiquitin C hydrolases 1 and 3, UCHL1/L3) related to regulation of penetration through the zona pellucida [50]. Differences in the expression of *RAB1B* could be related to the presumed increase of ubiquitinated protein aggregates prompted by the presence of spermatozoa in the oviduct.

Of particular interest was the over-expression of *TOR3A*. This gene showed the highest expression (11-fold change) in the oviduct of inseminated sows compared with the non-inseminated animals (Table 2). The pattern of expression of TOR3A protein in the oviduct (Fig 7) suggests a probable secretion of the protein to the oviductal lumen and implies a role during sperm-oviduct interactions and/or involvement in sperm-oocyte interactions. TOR3A presence into the oviductal fluid was confirmed by detection of the protein by Western blotting (Fig 8). Although unexpected, could be explained in the light of the new functional roles recently attributed to TORSIN A, including its involvement in the secretory pathway [51]. Whether the protein function in the oviduct is related to regulation of the inflammatory response or whether it is involved in the spermatozoa or early embryo physiology remains to be discovered. In our knowledge, this is the first time that *TOR3A* has been described in the oviduct and clearly expressed in the epithelial cells of the mucosa layer (Fig 7). However, further studies are necessary to perform to investigate the *TOR3A* function in the oviduct.

*B4GALT4* is another gene of this network, whose over-expression in oviductal tissue has not been reported previously, but it is known that B4GALT4 protein is expressed on the sperm surface of several mammals [52], possibly as a ligand to bind sperm to the zona molecule ZP3 in mouse [53]. In mouse cortical granules, it may play a role in blocking polyspermy [54].

Finally, Network 3 (Fig 5) associated functions are cell-to-cell signalling and interaction, and integrated 3 genes with the highest down-regulation level (*CYP51*, *PTH1R* and *TMOD3*; Table 3). *CYP51* down-regulation in the oviduct due to the presence of spermatozoa could be a consequence of the release of cholesterol, or its derivatives such as oxysterol, during the capacitation process of the sperm cells in this region at the time of the sampling. *PTH1R* and *TMOD3* (both with a fold change near to -3) are genes whose down-regulation could be related to the transport of gametes in the oviduct. *TMOD3* is expressed in motile endothelial cells, where it caps the pointed-ends of actin filaments and negatively regulates cell motility by controlling the turnover of F-actin [55]. *PTHR1* mediates the effects of PTH (parathyroid hormone) and PTH-rp (parathyroid hormone-related peptide) and has previously been shown to relax oviductal and uterine tissues from avian and mammalian species [56]. So, a reduction in *TMOD3* and *PTH1R* gene expression after insemination to facilitate fertilization and zygote residence in the oviduct for some hours could be expected.

Therefore, the data in the present study indicate a change in gene expression during male and female gametes encounter and interaction to form the zygote at the site of fertilization after a natural sperm selection within the female genital tract. These changes would indicate a modification of the environment preparing the oviduct for a successful fertilization and for an adequate zygote and embryo early stage milieu. New knowledge about the transcriptome modification around fertilization could be extended to the laboratory, adding new tools to increase IVF efficiency in mammals.

Overall, a key question is whether there is any relationship between inappropriate expression or failure of expression of specific oviduct genes and subsequent fertilization or embryo migration. Exploitation of gene knock-out models could be a fruitful way forward in this context.

## Supporting Information

S1 Fig Immunohistochemical quantification. Each bar shows the main intensity level of gray, where 0 is white and 255 black. Results are expressed as mean ± standard error. Different letters a,b,c in bars denote significant differences (p< 0.05). **A)** GSTA1 quantification. **B)** TOR3A quantification.
(TIFF)
