# Supplementary figures and images for: Oviductal Transcriptome Is Modified after Insemination during Spontaneous Ovulation in the Sow
Source: PLoS One. 2015 Jun 22;10(6):e0130128. doi: 10.1371/journal.pone.0130128 (PMC4476686; doi:10.1371/journal.pone.0130128)

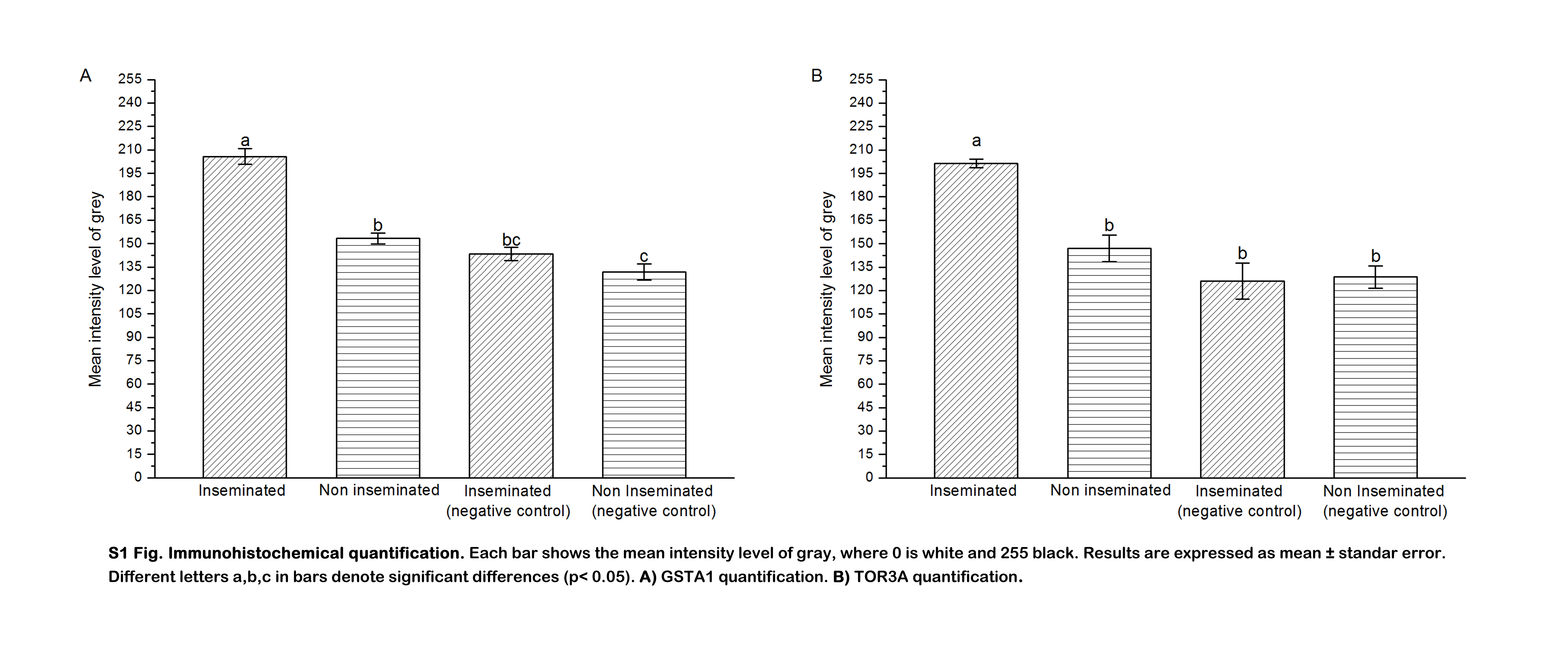

Supplement: S1 Fig — Each bar shows the main intensity level of gray, where 0 is white and 255 black. Results are expressed as mean ± standard error. Different letters a,b,c in bars denote significant differences (p< 0.05). A) GSTA1 quantification. B) TOR3A quantification. (TIFF) [file pone.0130128.s001.tiff]
